# Influence of Thermal Treatment on the Chemical and Structural Properties of Geopolymer Gels Doped with Nd_2_O_3_ and Sm_2_O_3_

**DOI:** 10.3390/gels10070468

**Published:** 2024-07-17

**Authors:** Miloš Nenadović, Sanja Knežević, Marija Ivanović, Snežana Nenadović, Danilo Kisić, Maja Popović, Jelena Potočnik

**Affiliations:** 1 Department of Atomic Physic, Vinča Institute of Nuclear Sciences, National Institute of the Republic of Serbia, University of Belgrade, Mike Petrović Alasa 12-14, Vinča, 11000 Belgrade, Serbia; dankisic@vin.bg.ac.rs (D.K.); majap@vin.bg.ac.rs (M.P.); jpotocnik@vin.bg.ac.rs (J.P.); 2 Department of Materials, Vinča Institute of Nuclear Sciences, National Institute of the Republic of Serbia, University of Belgrade, Mike Petrović Alasa 12-14, Vinča, 11000 Belgrade, Serbia; sanja.knezevic@vin.bg.ac.rs (S.K.); marija@vin.bg.ac.rs (M.I.); msneza@vin.bg.ac.rs (S.N.)

**Keywords:** geopolymer gels, Nd_2_O_3_, Sm_2_O_3_, open porosity, sorptivity, SEM, DRIFT, XPS

## Abstract

In this research, the influence of the thermal treatment of geopolymer gels at 300 °C, 600 °C and 900 °C when incorporated with 5% rare earth elements (REEs) in the form of (GP-Sm) Sm_2_O_3_ and (GP-Nd) Nd_2_O_3_ was investigated. Changes in the chemical and structural properties of the geopolymer gels during thermal treatment for 1 h were monitored. Physico-chemical characterization was performed using the following methods: diffuse reflectance infrared Fourier transform spectroscopy (DRIFTS), scanning electron microscopy with energy dispersive spectrometry (SEM-EDS), and X-ray photoelectron spectroscopy (XPS). Besides the characterization of the fundamental properties, some practical macroscopic properties were analyzed as well: sorptivity, open porosity, and Archimedean density. The stretching vibrations of Nd–O–Si and Sm–O–Si were confirmed at a value of around 680 cm^−1^and an Nd–O–Si absorption band at a higher value, together with the most dominant band of Si–O stretching vibration similar for all the samples. No significant chemical changes occurred. Structural analysis showed that for GP-Nd, the largest pore diameter was obtained at 900 °C, while for GP-Sm, the largest pore diameter was obtained at 600 °C. EDS confirmed the amount of dopant to be about 5%. X-ray photoelectron spectroscopy showed that for GP-Nd, the ratio of Si and Al changed the most, while for GP-Sm, the ratio of Si and Al decreased with increasing temperature. The contributions of both dopants in the GP-gel structure remained almost unchanged and stable at high temperatures. The atomic percentages obtained by XPS analysis were in accordance with the expected trend; the amount of Si increased with the temperature, while the amount of Al decreased with increasing temperature. The sorptivity and open porosity showed the highest values at 600 °C, while the density of both geopolymers decreased linearly with increasing temperature.

## 1. Introduction

Geopolymers represent a promising group of porous ceramic materials mostly known for their low energy consumption in the production process, with an accent on construction applications; they have a low price, and they are acceptable for the environment, with low emissions of CO_2_ [1,2]. The cement industry harms the atmosphere. The cement industry pollutes the atmosphere; thus, geopolymers are favored because of their unique purity. For these reasons, they are considered “green materials”. The activation of aluminosilicate materials, such as fly ash and metakaolin, using alkaline solutions to create new binders, represents a great challenge to the rapid increase in the potential use of industrial waste products [1,2,3] and the reduction in the harmful impacts of cement production. Geopolymers are most often obtained from aluminosilicate materials in the form of natural raw materials, but recently, they have been increasingly obtained from different types of waste materials [1,2,3]. These materials have been interesting for the scientific community lately because they have a lower carbon footprint than conventional cement binders. The application of thermal treatment contributes to the improvement in mechanical properties. This type of examination was carried out in this research. Exposing the geopolymer material to elevated temperatures leads to a complete structural change in the aluminosilicate matrix. Chemical bonds begin to break, which leads to the appearance of additional porosity and a cage structure. Such materials have the form of geopolymer gel foam, which has additional improved properties in the field of acoustic and thermal insulation [3]. Geopolymer materials have a very wide range of applications, and recent research has dealt with testing new properties of geopolymers in order to expand existing applications. In these studies, the open porosity, density, and sorptivity of geopolymer materials were monitored after thermal treatment. Earlier research confirmed by Kljajevic et al. [4] indicates that 900 °C is a significant transition temperature for metakaolin-based geopolymers, where the geopolymer partially melts and solidifies locally. A significant gap still exists at this temperature that separates the locally melted structures [5]. There are many experimental techniques that can qualitatively characterize aluminosilicates, but one stands out among them. Fourier transform infrared spectroscopy (FTIR) is probably the most affordable and easily available method for the characterization of geopolymers, and it has been frequently used to investigate their structural behavior [5,6,7,8,9,10]. Narasimharao and Ali confirmed that the most significant characteristic bands can be found at 1088 cm^−1^ and 1094 cm^−1^. These bands can be attributed to the asymmetric stretching of Al–O and Si–O bonds, respectively [11].

X-ray photoelectron spectroscopy (XPS) is a comprehensive analytical technique for quantitative and qualitative composition analysis that provides basic insights into information on chemical bonding. It can play an important role in the identification of different aluminosilicates based on the detailed analysis of the Al 2p and Si 2p spectral lines [4]. On the other hand, data interpretation is not always simple, mainly because of the inhomogeneous charging of non-conductive samples. Such phenomena lead to peak broadening and to difficulties in bond identification. Scanning electron microscopy (SEM) enables detailed insight into the microstructural behavior of geopolymers.

Water-permeable concrete is an environmentally friendly, green material with a composition that includes cementitious binders, water, and coarse aggregate. The potential usage of this type of material has several advantages, including storm-water management, reduced pavement noise, and pollutant control [12]. It is expected that cement is widely used as the main cementitious material. The main imperfections of cement, including significant CO_2_ emissions and great energy requirements [4,5,6,7,8,9,10,11], have prompted research in different directions [13] to develop alternative materials to produce a new type of concrete to face real factors and find the final solutions to these issues. In this way, geopolymer is a very suitable substitute material; it is an eco-friendly and sustainable material with a low carbon footprint, and it is obtained from the polycondensation of aluminosilicate solids activated by an aqueous solution of alkali silicate [4]. Geopolymer [14,15,16,17,18,19,20] has several advantages when compared to ordinary Portland cement (OPC): higher compressive strength, higher flexural strength, faster setting time, earlier strength development, lower CO_2_ emissions, and higher temperature resistance [21,22,23,24,25]. A novel water-permeable geopolymer with high strength and a high permeability coefficient was derived from fly ash, slag, and metakaolin [26,27,28,29,30,31,32,33]. Geopolymers doped with rare earth elements show interesting properties, such as improved magnetic, optical, catalytic, etc., properties [33]. Knežević et al. found that the incorporation of rare earth elements disrupts the basic structure of geopolymer properties in combination with thermal treatment [34]. In this research, thermally treated geopolymer was synthesized from metakaolin, obtained by thermal treatment of kaolin (Rudovci, Serbia), at 300 °C, 600 °C, and 900 °C. Monitoring the sorptivity, open porosity, and density of the material was conducted in detail. The main reason why Nd_2_O_3_ and Sm_2_O_3_ were chosen is because these two types of rare earth oxides complement each other. They have very great similarities because they represent rare earths and are of similar origin. On the other hand, they have an important difference, which is reflected in the photocatalytic sensitivity of Nd, while Sm does not show such properties. It was also interesting to examine how Nd and Sm affect the thermal stability of geopolymers. Oxides were used because of their stability at high temperatures, where thermal degradation is not desirable.

## 2. Results and Discussion

### 2.1. Diffuse Reflectance Infrared Fourier Transform Spectroscopy (DRIFTS)

**Figure 1:**
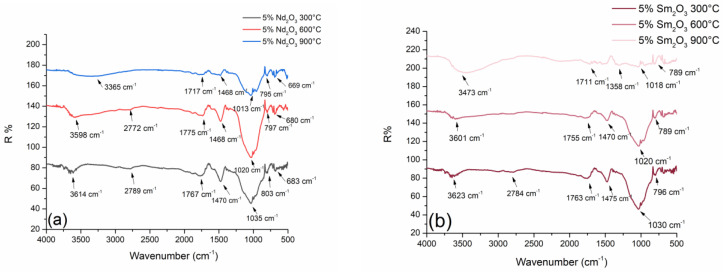
DRIFTS spectra of thermally treated GP: (**a**) Nd-doped; (**b**) Sm-doped.

The DRIFT analysis technique provides information about the energy of bond vibrations in materials so that specific groups, characteristic of some materials, can be identified. Figure 1 presents the chemical bonding of geopolymer molecules obtained from metakaolin using the DRIFTS technique.

In the far-infrared region, there are absorption bands that confirm the existence of a chemical bond between the dopant and the geopolymer base. The stretching vibrations of Nd-O-Si are found at a value of around 680 cm^−1^ and do not change significantly with the temperature of the thermal treatment. This confirms that neodymium remains chemically bound in the geopolymer structure (Figure 1a), and the Nd-O-Si absorption band slightly shifts to higher values. In the case of the geopolymer doped with samarium, the stretching vibrations of Sm-O-Si are found at a value of around 790 cm^−1^, whereas the temperature increase leads to the Sm-O-Si absorption band moving to lower values. Also, in the case of samarium, its presence in the geopolymer matrix was confirmed.

The most pronounced bands visible on all spectra are Si–O stretching vibration (1035 cm^−1^) and Si–O–Al vibration (803 cm^−1^) for Nd-doped geopolymer (Figure 1a), and the same absorption bands are found for Sm-doped geopolymer at 1030 cm^−1^ and 796 cm^−1^ (Figure 1b). The mentioned samples represent geopolymers treated at 300 °C and 600 °C. In the case of the presence of neodymium and samarium, the absorption bands do not change too much. The spectra are very similar, and the wave numbers are very close. This indicates that there was no significant chemical change in these samples. At higher values of wave numbers, one can observe the bending vibration of adsorbed water at 1775 cm^−1^ and 1755 cm^−1^, respectively. According to thermal treatment conditions at elevated temperatures, the additional adsorption of atmospheric CO_2_ occurs, which is confirmed by the occurrence of CO_2_ bands at values of around 2772 cm^−1^ and 2784 cm^−1^. 

In the near-infrared region, there are absorption bands of unstable hydroxides Nd(OH)_3_ and Sm(OH)_3_. They are found in all treated samples at around 3600 cm^−1^ (Figure 1a,b). What is most important for this research is monitoring the behavior of dopants in the geopolymer structure. Figure 1a,b clearly shows a slight transformation of the absorption bands of Nd(OH)_3_ and Sm(OH)_3_ with increasing temperature. Absorption bands widen up to the maximum treatment temperature at 900 °C (Figure 1a,b), where complete decomposition and disappearance of the chemical bond between Nd-OH and Sm-OH occur. The broad absorption band, which no longer confirms the existence of hydroxide, moves to slightly lower values of the wavenumber (3365 cm^−1^ and 3473 cm^−1^). In this research, DRIFTS confirmed that at high temperatures of the thermal treatment, the unstable Nd and Sm hydroxides are completely decomposed, and the dopant elements are incorporated into the geopolymer structure in a different form.

Some of the more important absorption bands, with their corresponding wave numbers and assignments, are given in Table 1 and Table 2. They represent the basic absorption bands of alumino-silicate materials with the addition of vibration of the hydroxide bond of Nd and Sm. Certainly, tabular values may vary depending on the spectrum-acquiring conditions, sample type, and many other parameters. However, these deviations must not be greater than a few tens of cm^−1^.

### 2.2. Scanning Electron Microscopy with Energy-Dispersive Spectrometry (SEM-EDS)

**Figure 2:**
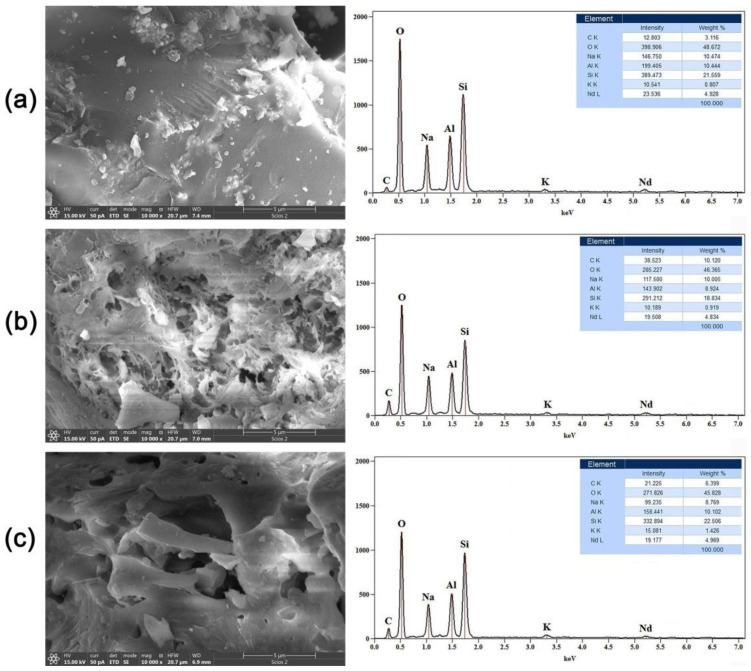
SEM images (**left side**) and corresponding EDS spectra (**right side**) of GP samples doped with 5% Nd and treated at (**a**) 300 °C, (**b**) 600 °C, and (**c**) 900 °C.

**Figure 3:**
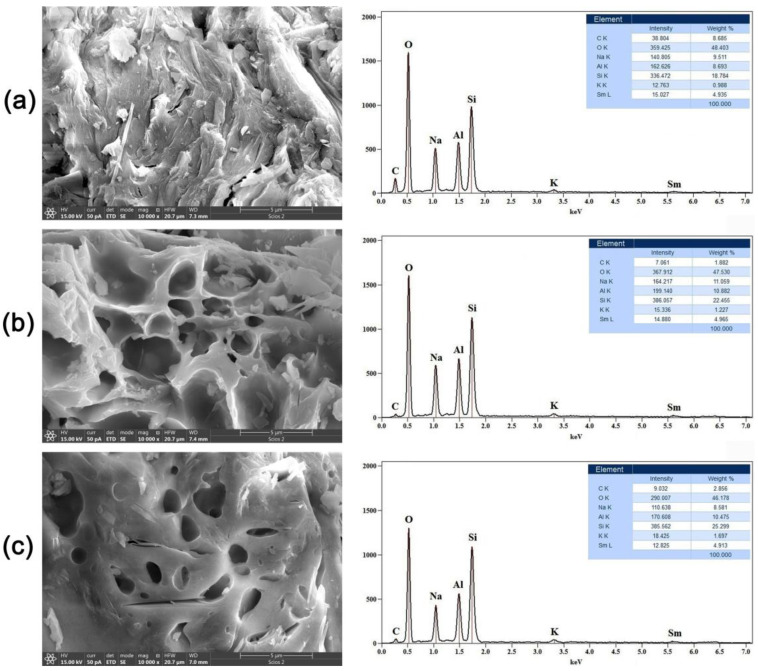
SEM images (**left side**) and corresponding EDS spectra (**right side**) of GP samples doped with 5% Sm and treated at (**a**) 300 °C, (**b**) 600 °C, and (**c**) 900 °C.

In order to analyze the morphology and elemental composition of the GP samples, SEM-EDS analysis was conducted, and the obtained results are shown in Figure 2 and Figure 3. The preliminary images of the GP samples doped with 5% Nd (Figure 2, left side) show that the morphology of thermally treated geopolymer has been changed.

As can be seen, after exposure to 300 °C (Figure 2a), the GP matrix forms a very dense structure with no visible cracks or pores. With an increase in the treatment temperature to 600 °C (Figure 2b), there is a considerable change in the structure of the geopolymer. One can notice the initiation of micro-cracks, both on the surface and in the bulk of the material. Furthermore, an increase in porosity is observed, which can be described as the formation of a sponge-like structure in the initial phase. A microphotograph of the third sample, which was treated at 900 °C, is shown in Figure 2c. It can be clearly seen that the small pores and micro-cracks have grown into large pores, indicating that the cavity-propagation phenomena have continued. The dimensions of the micro- and mesopores rapidly increased to several microns in diameter, which suggests that the given GP material became extremely porous and hollow in structure. The obtained results reveal that after thermal treatment at 900 °C, a geopolymer foam gel with unique characteristics was formed [32]. Finally, we can infer that for GP samples doped with neodymium, increasing the thermal treatment temperature results in an unambiguous increase in porosity, total active surface area, and volume.

Additionally, EDS analysis was used to evaluate and check the chemical composition of the samples, and the acquired results are shown in Figure 2 (right side). It can be seen that the spectra are almost identical, implying the homogeneity of the geopolymer samples used in this research. The most dominant lines belong to oxygen (O), silicon (Si), aluminum (Al), and sodium (Na). A small peak on the right side of the spectrum, around 5.3 eV, indicates the presence of Nd as a dopant.

SEM images of the geopolymer samples doped with samarium are shown in Figure 3 (left side). As can be noticed, the lowest treatment temperature of 300 °C (Figure 3a) causes the formation of a dense and compact geopolymer structure with visible cracks, which reaches its maximum during alkaline activation and the polymerization process.

Particular significance in this study is placed on the sample subjected to a treatment temperature of 600 °C (Figure 3b). The presence of extensive signs of cavity propagation and large pores is clearly visible, which means that at this temperature, an accelerated transformation reaction leads to the conversion of the dense GP gel to a very porous foam structure. If we look at the sample in Figure 3c, one can see that at the maximum temperature of 900 °C, the pores gradually close, and the geopolymer foam loses its porosity and openness. Based on the SEM images, it can be seen that for GP doped with samarium, the maximum pore propagation and opening of the porous structure occurs at 600 °C, in contrast to GP doped with neodymium, where pore sizes directly correspond to temperature. A preliminary conclusion can be reached that Sm in the GP structure lowers the energy and temperature required for the formation of geopolymer foam.

The corresponding EDS spectra, shown in Figure 3 (right side), provide insight into the general chemical composition of the obtained samples. As in the case of Nd-doped samples, the main lines correspond to O, Al, Si, and Na, while the peak positioned at 5.6 eV confirms the presence of samarium at approximately 5%. This information confirms that the presence of samarium is responsible for the lower GP foam gel forming temperature.

### 2.3. X-ray Photoelectron Spectroscopy (XPS)

**Figure 4:**
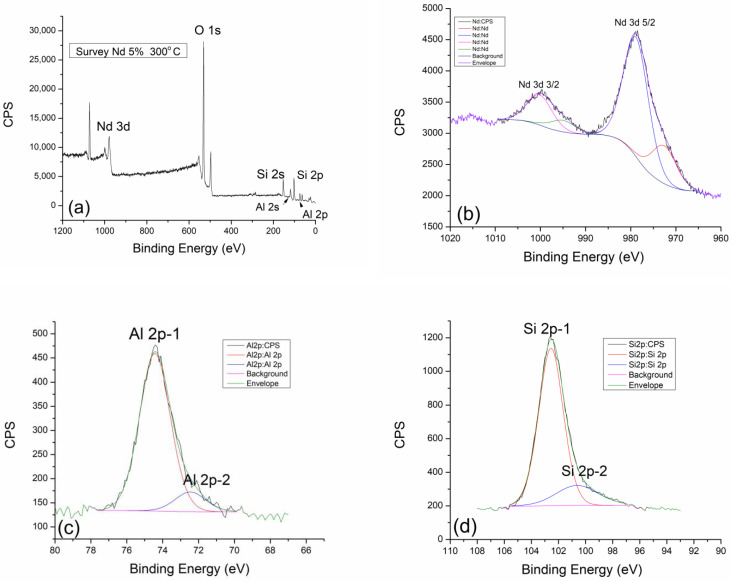
XPS analysis of GP with 5% Nd treated at 300 °C: (**a**) Survey XPS spectrum, (**b**) Nd 3d, (**c**) Al 2p, and (**d**) Si 2p regions.

Figure 4a shows the survey spectrum of geopolymer thermally treated at 300 °C. The most dominant spectral line belongs to oxygen O 1s, while very pronounced peaks for aluminum Al 2p and silicon Si 2p are found at characteristic positions for these elements. Neodymium appears on this spectrum as expected at a binding energy of around 1000 eV, where its peaks are divided into two dominant ones. It is very important to emphasize that the spectral line of carbon is minimized or does not exist. This means that the thermally treated geopolymer is carbon-free, material carbon-free, and suitable for all future green applications. Compared with thermally untreated geopolymers, where the carbon peak is clearly visible, after thermal treatment, the carbon almost completely leaves the structure and composition of the geopolymer [13]. 

High-resolution XPS spectra of Nd 3d, Al 2p, and Si 2p lines and Si 2p lines, together with the results of the corresponding fits, are shown in Figure 4b–d, respectively. Nd 3d line is a doublet positioned at 979.1 eV and 1000.5 eV, which belongs to Nd 3d5/2 and Nd 3d3/2, respectively. The first and more dominant contribution can be found at a binding energy of 972.8 eV and 979.1 eV (Nd 3d 5/2) and can be assigned to the existence of an equilibrium mixture of Nd(OH)_3_ + Nd_2_O_3_ as a consequence of alkaline activation. The second contribution, known as a satellite peak, gives the spectral line on 995.1 eV and 1000.5 (Nd 3d 3/2) and can be attributed to unreacted Nd_2_O_3_ as a starting dopant. After thermal treatment at the lowest temperature, we come to the conclusion that the amount of reacted and unreacted Nd_2_O_3_ is 2:1 [33].

Figure 4c gives an insight into the distribution of aluminum and its behavior after treatment at 300 °C. Two dominant spectral lines, Al 2p-1 (74.4 eV) and Al 2p-2 at 72.5 eV, are clearly visible in the graph. The more dominant Al 2p-1 peak can be attributed to pure Al_2_O_3_, while the relatively less pronounced contribution of Al 2p-2 refers to the existing phase of incompletely reacted aluminum in the form of CaAl_2_O_4_.

The detailed spectrum of silicon is shown in Figure 4d, where silicon behaves very similarly to aluminum. The graph clearly shows two dominant contributions of Si 2p-1 at 102.9 eV and a weaker contribution of Si 2p-2 at 100.6 eV. The Si 2p-1 peak at 102.9 eV can be attributed to the SiO_2_ and Al_2_OSiO_4_ dominant phase. Another contribution of Si 2p-2 on 100.6 eV corresponds to the molecular sieve (zeolite 3A) of NaAl_2_SiO_4_ [13]. By analyzing the thermally treated geopolymer at 300 °C, we can compare it with the thermally untreated one [13] and see that there are no significant differences in chemical behavior, as well as in terms of newly formed phases.

**Figure 5:**
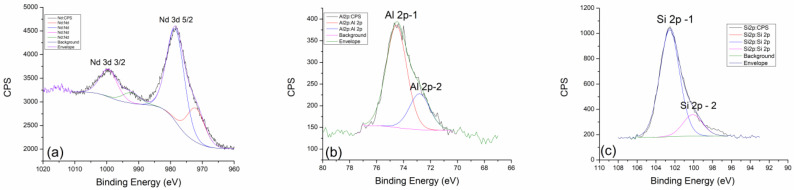
High-resolution XPS spectra taken from GP with 5% Nd treated at 600 °C: (**a**) Nd 3d, (**b**) Al 2p, and (**c**) Si 2p core-level lines with corresponding fits.

Figure 5 shows the detailed spectra of neodymium Nd 3d line, aluminum Al 2p line, and silicon Si 2p line of the geopolymer sample treated at 600 °C. As for neodymium, the peak positions of the Nd 3d 5/2 and Nd 3d 3/2 contributions remained unchanged. The ratio of the dominant and weaker contribution also did not change with temperature. This speaks to the thermal stability of neodymium and its inertness at elevated temperatures.

However, aluminum in this sample shows a completely different behavior. The contribution of Al 2p-2 at 72.5 eV becomes more pronounced and is not as small as in the case of treatment at 300 °C. The more dominant contribution of Al 2p-1 at 74.4 eV remains almost unchanged, which indicates the stability of the equilibrium of SiO_2_ and Al_2_OSiO_4_, while the elevated temperature of 600 °C additionally favors the reaction of the formation of the CaAl_2_O_4_ phase.

As aluminum and silicon behave similarly in the geopolymer, the less pronounced contribution of Si 2p-2 became more pronounced in the silica mixture, where the formation of the amorphous phase of the molecular sieve and NaAl_2_SiO_4_ was favored as the treatment temperature increased.

**Figure 6:**
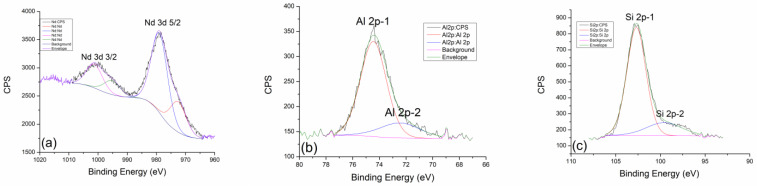
High-resolution XPS spectra taken from GP with 5% Nd treated at 900 °C: (**a**) Nd 3d, (**b**) Al 2p, and (**c**) Si 2p core-level lines with corresponding fits.

After thermal treatment of geopolymer at 900 °C, it can be concluded that neodymium definitely shows resistance to elevated temperatures. The positions of the spectral lines Nd 3d 5/2 and Nd 3d 3/2 remain exactly the same as in the previous cases (Figure 6a). Also, the ratio of the areas under the peaks remains unchanged. The balance between Nd(OH)_3_ and Nd_2_O_3_ phases remains almost constant in the entire range of temperatures to which the samples were exposed [33]. In this way, neodymium confirmed its role as a useful dopant because it behaves very well at all temperatures and does not affect the basic geopolymer composite much. In the case of thermal treatment at 900 °C, aluminum loses the intensity of the second Al 2p-2 contribution at 72.5 eV, where the decomposition of the CaAl_2_O_4_ phase undoubtedly occurs. This leads us to the conclusion that by curing at high temperatures, the reaction of thermal decomposition will be favored toward the formation of the most stable and dominant phase of SiO_2_ in the form of quartz.

**Figure 7:**
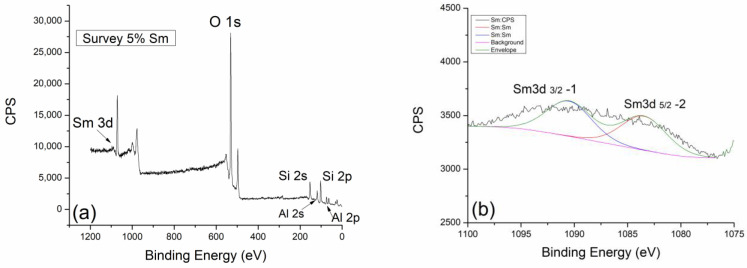

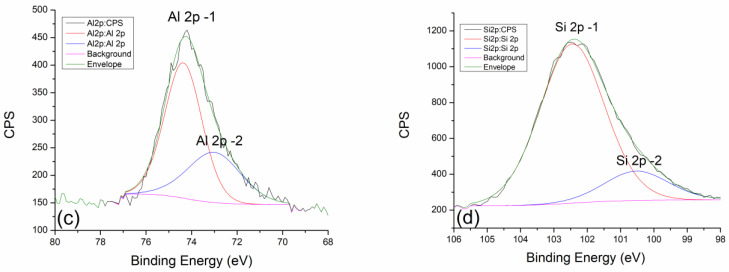
XPS analysis of GP with 5% Sm treated at 300 °C: (**a**) survey XPS spectrum, (**b**) Sm 3d, (**c**) Al 2p, and (**d**) Si 2p regions.

Figure 7a shows the survey spectrum of geopolymer thermally treated at 300 °C doped with 5% Sm_2_O_3_. The oxygen peak is located at the standard energy value (between 531.3 and 535.8 eV), and it was taken as the reference peak with which we analyzed the other contributions. Also, the positions of the Al 2p and Si 2p spectral lines are expected and marked in Figure 7a. In the high-energy part of the spectrum (around 1000 eV), there is the spectral line of samarium, Sm 3d. What is again extremely important to point out is that the spectral line of carbon has completely or almost completely disappeared. This again speaks in favor of the fact that with the thermal treatment, we definitely form a carbon-free material, regardless of the type of dopant. The other survey spectra at 600 °C and 900 °C look almost identical, so for that reason, they will not be shown.

The presence of samarium as a dopant, which was confirmed and shown on the survey spectrum, is analyzed in detail in Figure 7b. For the purposes of the analysis, the Sm 3d 5/2 spectral line was taken, which has two almost identical contributions. The first Sm 3d 5/2-1 contribution is located at 1091.3 eV, while the second Sm 3d 5/2-2 is located at 1083.8 eV. The peak at the higher value of the binding energy refers to the equilibrium mixture Sm_2_O_3_ + Sm (OH)_3_, while the one at the lower value refers to the initial dopant Sm_2_O_3_.

**Figure 8:**
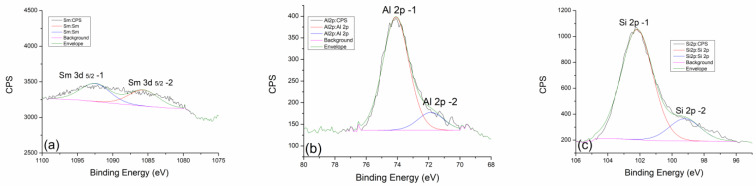
High-resolution XPS spectra taken from GP with 5% Sm treated at 600 °C: (**a**) Nd 3d, (**b**) Al 2p, and (**c**) Si 2p core-level lines with corresponding fits.

**Figure 9:**
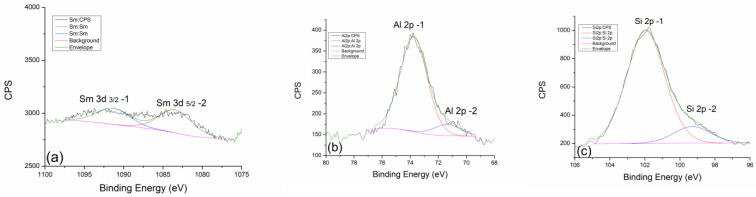
High-resolution XPS spectra taken from GP with 5% Sm treated at 900 °C: (**a**) Nd 3d, (**b**) Al 2p, and (**c**) Si 2p core-level lines with corresponding fits.

In the case of thermal treatment at 300 °C, this contribution ratio does not change significantly. Even at higher temperatures, the contribution ratios of Sm 3d 5/2-1 and Sm 3d 5/2-2 do not change drastically (Figure 8a). Only in the case of the highest treatment temperature at 900 °C is there a small jump in the intensity of the 3d 5/2-2 contribution in Figure 9a. This indicates an increase in the amount of pure Sm_2_O_3_ compared to the equilibrium mixture. Perhaps it is more correct to say that at very high temperatures, the equilibrium mixture disappears, and the reaction is favored toward the direction of the initial Sm_2_O_3_ formation. In the final phase of the thermal treatment, we expect that there will be complete decomposition and disappearance of the samarium–hydroxide phase.

The thermodynamically more stable oxide phase (Sm_2_O_3_) of samarium certainly remains in the composition of the geopolymer.

In the case of aluminum and silicon, whose behavior we follow very carefully, we can say with great certainty that they are almost identical to samples doped with neodymium. Al 2p and Si 2p spectral lines of geopolymer doped with neodymium at 300 °C (Figure 7c,d) show the expected behavior trend of a less pronounced contribution of Al 2p-2 and Si-2p-2. With an increase in temperature to 600 °C (Figure 8b,c), there is a slight decrease in the contribution of Al 2p-2 and Si 2p-2 lines, while at the maximum temperature of 900 °C, a greater decrease in the intensity of these spectral lines can be observed. The explanation for this trend is very similar to the one in the case of neodymium. For this reason, we could conclude that dopants such as Nd and Sm show great inertness in terms of reacting with the geopolymer as well as with its intermediate phases in the process of alkaline activation and during thermal treatment.

In this regard, through further analysis of the numerical data obtained by the XPS method, the atomic percentages for silicon and aluminum were calculated, as well as their change, depending on the temperature of the thermal treatment (Table 3 and Table 4). Table 3 shows the change in atomic percentages with temperature for the neodymium-doped geopolymer. A certain regularity can be observed immediately because the amount of aluminum decreases with temperature from 10.06 at% to 8.43 at%. In the case of silicon, the completely opposite trend of behavior occurs.

The atomic percentages of silicon increase from 20.78 at% to 22.39 at%. If we look a little more carefully at the difference, we will see that the decrease in the amount of aluminum Al 2p is 1.63 at%, while the increase in Si 2p is 1.61 at%.

In the case of geopolymer doped with samarium, something similar but not identical happens. The atomic percentages of aluminum do not change continuously, but in the end, an overall decreasing trend can be observed (Table 4). The largest decrease in the amount of aluminum occurs at 600 °C, but even after the maximum temperature, the amount of Al 2p remains reduced by 0.34 at%. The maximum difference in this case is 0.57 at%. The behavior of Si 2p is identical to that of the sample with Nd; a constant increase in atomic percentages with increasing temperature is noticeable. The maximum difference in atomic percentages for this sample is 1.43 at%. Based on the obtained data on atomic percentages from the XPS analysis, one can say that with the increase in the thermal treatment temperature, the total amount of Al 2p decreases while the total amount of Si 2p increases. With geopolymer doped with samarium, we can definitely conclude that the percentage differences are reduced compared to the sample doped with neodymium. Neodymium probably did not affect the thermal losses of Al compounds in the geopolymer, while samarium somewhat thermally stabilized the more unstable Al phase and reduced the total losses, even in the case of the silicon’s more stable phase.

### 2.4. Sorptivity, Open Porosity, and Archimedean Density

**Figure 10:**
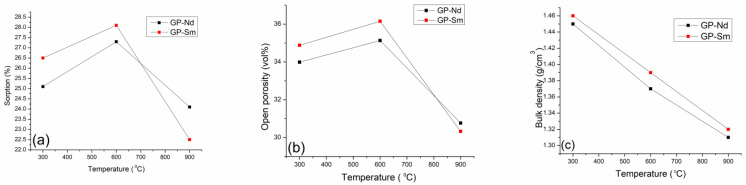
Comparative analysis of thermally treated geopolymers (GP): (**a**) sorptivity, (**b**) open porosity, and (**c**) bulk density.

Within this section, comparative analyses of three parameters that are essential for a complete understanding of the behavior of thermally treated geopolymers were performed. Sorption shows an increasing trend for temperatures of 300 °C and 600 °C, while at the highest temperature, there is a drastic drop in both sorption and open porosity (Figure 10a,b). This can be related to the thermal decomposition of the basic structure of the geopolymer and the disappearance of the basic chemical bonds in the geopolymer. In that case, there is a reduction in the possibility of receiving water into its structure and an obvious drop in open porosity.

All this was followed by a constant drop in the bulk density (Figure 10c), where a constant, and in the case of GP-Sm, a linear drop in density is observed. In the case of GP-Nd, there is a slight deviation, but it can also be approximated by a linear dependence on temperature. Slightly higher values of bulk density of GP-Sm compared to GP-Nd come from the higher density of Sm_2_O_3_ (8.35 g/cm^3^) compared to Nd_2_O_3_, whose density is 7.24 g/cm^3^.

Finally, we can conclude that at a temperature of 900 °C, reactions take place that degrade the geopolymer structure and turn it into a geopolymer gel foam with special structural properties. This property is first of all reflected in the reduced density, but not the reduced resistance to environmental conditions. The density of geopolymer foam, which is somewhere around 1.30 g/cm^3^, can significantly increase the prospect of these light but stable materials being additionally used in further applications.

## 3. Conclusions

This research has investigated the influence of the thermal treatment of geopolymers at 300 °C, 600 °C, and 900 °C incorporated with 5% rare earth elements (REEs) in the form of Sm_2_O_3_ and Nd_2_O_3_. Various changes in the chemical and structural properties of geopolymer gels during thermal treatment were observed. DRIFT revealed the specific change in the spectra obtained for GP treated at 900 °C. The broad peak appears around 3623 cm−1 as a consequence of the thermal decomposition of the Nd-OH and Sm-OH chemical bonds. Microscopic analysis revealed that for the Nd_2_O_3_-doped GP, the maximum pore opening is reached for thermal treatment at 900 °C. When doping GP with Sm, an unexpected deviation occurred, and the maximum pore diameters appeared at 600 °C. It can be concluded that samarium ions contributed to lowering the temperature and activation energy needed to obtain the porous GP gel foam material. It can be said that samarium in GP behaves as a kind of catalyst, while the presence of neodymium does not show any special influence. These claims are further substantiated by XPS analysis, which confirms that the amount of neodymium and samarium does not change almost at all with increasing treatment temperature. What is subject to change are the amounts of aluminum and silicon. This research confirmed that the amount of aluminum decreases with temperature while the amount of silicon increases slightly. The calculated values of sorptivity and open porosity show their maximum at 600 °C, regardless of the influence of dopants. Based on these results, it could be said that temperature plays a more important role in the formation of porous geopolymer and that the presence of samarium additionally accelerates the process. The total density decreases constantly with an increase in the thermal treatment temperature. The average drop in density of about 0.15 g/cm^3^ still affects the production of even lighter and porous geopolymers. All this research leads in the direction of obtaining resistant, light, and porous geopolymer gel structure foams. The tested samples are good candidates for this type of material.

## 4. Materials and Methods

Geopolymer (GP) was synthesized from metakaolin (MK) with 5% Nd and Sm, calculated to molecular weights of Nd_2_O_3_ and Sm_2_O_3_. Geopolymer gels were produced by the reaction of metakaolin with a sodium silicate activating solution. The alkali activation solution consisted of a sodium hydroxide solution (Sigma-Aldrich, St. Louis, MO, USA) and a sodium silicate solution (Interhem company, Belgrade, Serbia). NaOH solutions were prepared in 12 M concentration. The volume ratio of the NaOH solution to the Na_2_SiO_3_ solution was 1.5. Liquid-to-solid phase ratio was approximately 1.0. The alkali activation solution and precursors were mixed for a short time, poured into molds, covered, and left at room temperature for 48 h. The samples were placed in an oven at 60 °C for 24 h in molds with cover. After drying in the oven, the obtained samples were left for 28 days at room temperature. Geopolymer concretes are a new type of concrete synthesized from metakaolin, fly ash, diatomaceous earth, and bentonite activated with an alkaline activator [13]. Earlier research confirmed that the incorporation of rare earth elements (Nd and Sm) into the geopolymeric matrix results in a geopolymeric gel structure material with better physical and chemical characteristics. The aim of this research is to examine the effect of thermal treatment at 300 °C, 600 °C, and 900 °C on open porosity, sorptivity, and Archimedean density.

### Characterization Techniques

In this research, the following experimental techniques were used: diffuse reflectance infra-red Fourier transform spectroscopy (DRIFTS), scanning electron microscopy with energy dispersive spectrometry (SEM-EDS), and X-ray photoelectron spectroscopy (XPS). After 28 days, open porosity, sorptivity, and Archimedean density were calculated.

Drift spectra were obtained using the PerkinElmer FTIR spectrometer Spectrum Two. Approximately 5% of samples were dispersed in oven-dried spectroscopic grade KBr with a refractive index of 1.559 and particle size of 5–20 μm. The spectra were scanned at 4 cm^–1^ resolution and collected in the mid-IR region from 4000 to 400 cm^–1^.

The morphology and structure of thermally treated geopolymers were analyzed by field emission scanning electron microscopy (SEM) using FEI SCIOS 2, Dual Beam electron microscope, operated at 15 kV. XPS analysis was performed using a SPECS instrument for detailed chemical composition characterization using X-ray-induced photoelectron spectroscopy. Photoelectron emission was excited by monochromatic Al Kα line with a photon energy of 1486.67 eV. Survey spectra were recorded in the fixed analyzed transmission mode with a pass energy of 40 eV, an energy step width of 0.5 eV, and a dwell time of 0.2 s. Detailed spectra of the main photoelectron lines were taken at pass energy of 20 eV (FAT 20), an energy step of 0.1 eV, and a dwell time of 2 s. Charging compensation was performed using an electron flood gun and the constant current and voltage.

Sorptivity and open porosity test was carried out according to Hall (1989). In this test, samples were dried in the oven at 105 °C to constant mass. After cooling, the bottom surface of the sample was cut, and this cut surface was made to contact with water. The test was carried out by measuring the weight gain of the samples at the set time intervals of 5 min, 10 min, 30 min, 1 h, 2 h, 3 h, and 4 h.

Archimedean density was determined using standard procedure at the constant mass (dry mass) of the samples. Samples were placed in the oven and dried, and the mass of the samples was determined. After a while, samples were dried in a dryer at a temperature of 100 to 110 °C (the most ideal 105 °C) for at least 24 h. After drying, samples were cooled in dry air (preferably in a desiccator) to a temperature of 20 to 25 °C. Subsequently, the mass of the samples was determined.

## Figures and Tables

**Table 1:** Values of wave numbers and their assignment of Nd-doped GP.

Values of Wavenumbers (cm^−1^)	Assignment
3614	Nd-OH
2798	adsorption of atmospheric CO_2_
1767	vibration deformation of adsorbed water
1470	adsorption of atmospheric CO_2_
1035	asymmetric vibration Si-O-Al
796	symmetrical vibrations Si-O-Al
683	stretching vibrations Nd-O-Si

**Table 2:** Values of wave numbers and their assignment of Sm-doped GP.

Values of Wave Numbers (cm^−1^)	Assignment
3623	Sm-OH
2784	adsorption of atmospheric CO_2_
1763	vibration deformation of adsorbed water
1475	adsorption of atmospheric CO_2_
1030	asymmetric vibration Si-O-Al
796	stretching vibrations Sm-O
669	bending vibrations Sm-O-H
561	stretching vibrations Sm-O-Si

**Table 3:** Atomic percentages (at%) of aluminum and silicon in Nd-doped thermally treated geopolymer samples obtained by XPS analysis.

GP Nd 300 °C	(at%)	GP Nd 600 °C	(at%)	GP Nd 900 °C	(at%)
Al 2p	10.06	Al 2p	8.77	Al 2p	8.43
Si 2p	20.78	Si 2p	21.45	Si 2p	22.39

**Table 4:** Atomic percentages (at%) of aluminum and silicon in Sm-doped, thermally treated geopolymer samples obtained using XPS analysis.

GP Sm 300 °C	(at%)	GP Sm 600 °C	(at%)	GP Sm 900 °C	(at%)
Al 2p	9.49	Al 2p	8.92	Al 2p	9.15
Si 2p	20.08	Si 2p	20.82	Si 2p	21.51

## Data Availability

The data presented in this study are openly available in article.
